# Priors Engaged in Long-Latency Responses to Mechanical Perturbations Suggest a Rapid Update in State Estimation

**DOI:** 10.1371/journal.pcbi.1003177

**Published:** 2013-08-15

**Authors:** Frédéric Crevecoeur, Stephen H. Scott

**Affiliations:** 1 Centre for Neuroscience Studies, Queen's University, Kingston, Canada; 2 Department of Biomedical and Molecular Sciences, Queen's University, Kingston, Canada; University College London, United Kingdom

## Abstract

In every motor task, our brain must handle external forces acting on the body. For example, riding a bike on cobblestones or skating on irregular surface requires us to appropriately respond to external perturbations. In these situations, motor predictions cannot help anticipate the motion of the body induced by external factors, and direct use of delayed sensory feedback will tend to generate instability. Here, we show that to solve this problem the motor system uses a rapid sensory prediction to correct the estimated state of the limb. We used a postural task with mechanical perturbations to address whether sensory predictions were engaged in upper-limb corrective movements. Subjects altered their initial motor response in ∼60 ms, depending on the expected perturbation profile, suggesting the use of an internal model, or prior, in this corrective process. Further, we found trial-to-trial changes in corrective responses indicating a rapid update of these perturbation priors. We used a computational model based on Kalman filtering to show that the response modulation was compatible with a rapid correction of the estimated state engaged in the feedback response. Such a process may allow us to handle external disturbances encountered in virtually every physical activity, which is likely an important feature of skilled motor behaviour.

## Author Summary

It is commonly assumed that the brain uses internal estimates of the state of the body to adjust motor commands and perform successful movements. A problem arises when external disturbances deviate the limb from the ongoing task. In such cases, the estimated state of the body must be corrected based on sensory feedback. Because neural transmission delays can destabilize feedback control, an important challenge for motor systems is to correct the estimated state as quickly as possible. In this paper, we tested whether such a rapid correction is performed following mechanical loads applied to the upper limb. Our results indicate that long latency responses (∼50–100 ms) exhibit knowledge of the relationship between the delayed sensed joint displacement and the current state of the limb at the onset of the motor response. Importantly, this knowledge can be adjusted from one perturbation response to the next, should a distinct perturbation profile be experienced. These results suggest that a correction of state estimation is performed within the limb rapid-feedback pathways, allowing fast and stable feedback control.

## Introduction

Neural transmission delays present a major challenge because the brain cannot directly use sensory feedback to guide motor actions. In order to compensate for feedback delays, the brain must build internal models of the dynamical interaction between the body and the environment, including sensory and motor prediction mechanisms. On the one hand, motor predictions use forward models to convert motor commands into estimates of the state of the body [1]. On the other hand, sensory prediction uses current sensory data to anticipate future events in various contexts. For instance, with enough sensory information, humans can easily anticipate the re-appearance of a visual target that is briefly occluded [2], [3]. Another example is the anticipatory scaling of grip-force with expected load constraints estimated from fingertip sensory encoding prior to the object lift [4].

An important question is whether the motor system uses similar processes to guide feedback responses to mechanical perturbations. Indeed, perturbation loads applied on the upper limb evoke very quick, task-related responses (long-latency, ∼50 ms) [5]. Because delays as short as tens of milliseconds can destabilize motor corrections, we hypothesize that a rapid sensory prediction is performed to update the estimated state of the limb. This problem has received little attention because previous modeling studies have often assumed that delays are equivalent to instantaneous but noisier signals [6]–[10]. This approach is partially justified by the fact that increasing the feedback delay or the feedback noise similarly increases the variability of unperturbed behaviour [11], but it is inadequate when abrupt perturbations induce large amounts of joint displacement. Also, previous work suggesting the presence of such a sensory-based prediction did not test directly whether such a process was engaged on a time scale corresponding to long-latency delays [12]–[14]. Thus, it remains unknown how quickly the internal estimation is corrected and used in the motor response.

The words ‘sensory prediction’ and ‘motor prediction’ have been often used in the literature to designate the same process, which is the prediction of the consequences of motor commands based on efference copy and internal forward models [15]. In the present paper, we make a distinction between the prediction based on forward models, which we referred to as ‘motor prediction’, and the process under investigation, which converts delayed sensory data into estimates of the actual state. We refer to this process as ‘sensory prediction’, in the sense that it does not rely on efference copy of the motor command.

**Figure 1:**
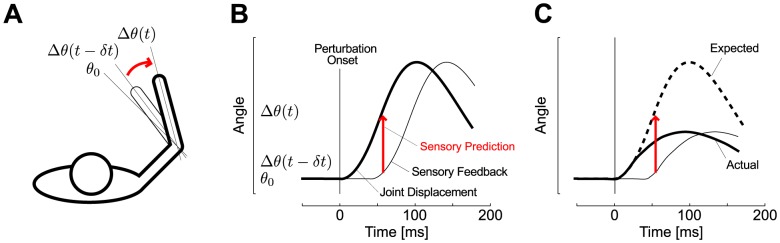
Correction of state estimation following a perturbation. A: Overhead sketch of a perturbation evoked displacement relative to the prescribed joint location (*θ_0_*). The initial change in joint angle following a perturbation is sensed after some delay (thin trace, *Δθ(t−δt)*). A correction of the actual change in joint angle (thick trace, *Δθ(t)*) involves a prediction based on the available sensory data combined with an internal model of the perturbation (red arrow). B: Schematic representation of the sensory prediction on the joint displacement plotted as a function of time (numbers are for illustration). The sensory prediction (red arrow) estimates the present state of the limb (thick trace) based on delayed sensory feedback (thin trace) and internal assumptions about the perturbation profile. C: Illustration of overestimation resulting from updating the current state estimate based on an expected movement profile (dashed trace) that follows the same initial displacement as the actual one but diverges during the time interval corresponding to the feedback delay (solid trace).

In theory, sensory prediction is expected if optimal state estimation is performed while taking feedback delays into account (Kalman filter). In this framework, the present state of the limb (Figure 1 A, *Δθ(t)*) is corrected based on the delayed sensory signal available at time *t* (*Δθ(t−δt)*) combined with an internal model of how the perturbation affects limb motion (Figure 1 A–B). This model makes two important predictions: (i) perturbations of varying amplitude should be easily handled as long as their profile corresponds to the participants' internal model; (ii) corrective responses for unexpected time-varying perturbations would be initially biased towards responses for the expected ones. We tested these predictions by manipulating the probabilities of different perturbations applied on the upper limb. The odd perturbations shared similar initial force profiles but changed rapidly (Figure 1 C), causing unexpected variations in the joint motion that should impact the motor response.

In agreement with the model, we show that responses to step perturbations scaled with the step magnitude, regardless of whether changes in magnitude were expected or not. In contrast, initial responses to other unexpected perturbation profiles matched the response for the expected perturbation profile, suggesting that internal models are engaged in these rapid corrective responses. These priors started to influence the motor response within the long-latency time window (∼50–100 ms). Changes in long-latency responses correlated with the expected relationship between the initial joint displacement and the true state of the limb at the onset of the motor response as predicted by simulations using optimal state estimation. Altogether, our results suggest that state estimation guides long-latency motor responses to mechanical perturbations.

## Results

### State Estimation Stabilizes Feedback Responses

**Figure 2:**
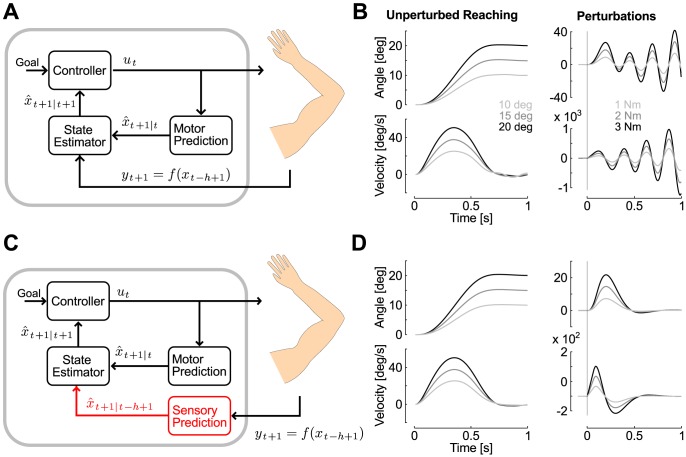
Computational models and simulations. A: Computational model based on a state estimator that ignores feedback delays and directly integrates sensory feedback with prior beliefs about the body state. The controller outputs a motor command (*u_t_*) specified by the behavioral goal and motor costs. The forward dynamic model (Motor Prediction) predicts the consequence of the descending motor command (Predicted Consequences, 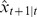). The feedback signal (*y_t+1_*) is a function of the delayed state vector expressed in *f(x_t−h+1_)*, where *h* represents the feedback delay in number of sample times (see Methods). B: Simulation of the model corresponding to panel A on the control of a single joint actuator during unperturbed reaching movements of varying amplitudes (left) and following perturbation loads of three selected magnitudes (right). Displays are the joint angle (top) and angular velocity (bottom). Time 0 corresponds to the onset of the reaching movement, or the perturbation onset (solid line). C: Full model with the sensory predictor highlighted in red. Following a perturbation, the output of the motor prediction still indicates that the joint displacement is zero, but the sensory prediction corrects the estimate of the joint state based on maximum-likelihood principle. This is illustrated by the conditional expectation about the present state, given delayed sensory information (Predicted Current State, 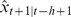) D: Same as B with the control and state estimation corresponding to panel C.

The effect of feedback delays on motor performances have been studied in the context of voluntary movement control, with feedback delays typically greater than those characterizing rapid motor responses to perturbations (for instance, visuomotor delays are >100 ms) [14], [16], [17]. Although responses to mechanical perturbations can be quicker, delays of the order of tens of milliseconds can also destabilize feedback responses. The effect of feedback delays is illustrated in Figure 2 with simulations from a feedback controller that must keep a joint at a prescribed angle with two distinct state estimators (see Methods). In the first case (Figure 2 A), the state estimator directly weighted the current feedback signal with the internal prior, taking only the variances of each signal into account and ignoring the feedback delay (δt = 60 ms, see Methods). This control mechanism could generate stable reaching movements of varying amplitude, but it was prone to instability in the presence of external perturbations (Figure 2 B). We observed numerically that decreasing the weight of the feedback signal by increasing sensory noise could stabilize the process because the controller relies less heavily on sensory feedback. However, we could reject this possibility because the resulting feedback corrections were too slow and incompatible with human motor behaviour. Note that the simulations presented in Figure 2 B were obtained after decreasing the weight of sensory feedback by a factor of 20 relative to the parameters used otherwise. Stability issues can also be encountered with control processes based a Smith predictor [16], because these controllers are extremely sensitive to mismatch between the internal model and the actual plant [18]. However, prediction errors are ubiquitous in biological motor control because of the multiple sources of neural noise [19], and the presence of external disturbances.

In order to produce stable and accurate feedback responses, we suggest that motor systems rely on optimal state estimation while taking feedback delays into account (Figure 2 C, Methods). The resulting controller generated stable reaching movements as well as feedback responses to the same perturbation loads (Figure 2 D). Such a rapid correction involves a prediction based on the actual sensory data combined with an internal model (or prior) about the effect of the perturbation on the limb (Figure 2 C and D). Observe that this mechanism is distinct from the usual motor prediction because there is no causal relationship between the motor command and the motion of the body. If participants rely on a similar mechanism, the theory predicts that internal models of the perturbation profiles must be engaged at the onset of the motor response. This prediction was confirmed by the experiments presented below. We first emphasize that internal priors modulate long-latency responses to perturbations (Experiment 1). The second experiment shows a trial-by-trial adaptation of these priors to changes in perturbation profiles. Finally, we present two control experiments confirming that these priors do not depend on the muscle pre-activation (Experiment 3), and are specific to the shape, and not the amplitude, of the perturbation loads (Experiment 4).

### Experiment 1

**Figure 3:**
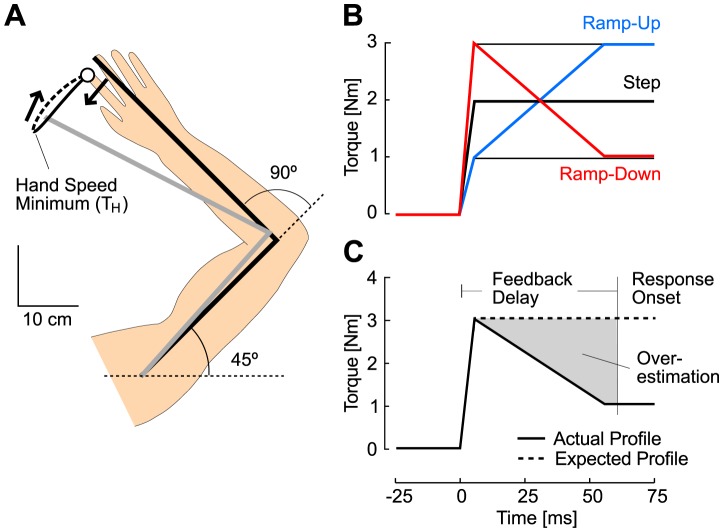
Procedures and perturbations. A: Overhead representation of the initial joint configuration and of a typical perturbation related movement. The initial joint configuration is shown in black. Hand path from perturbation onset until the first hand-speed minimum (T_H_) is represented in solid trace. The remaining portion of the corrective movement is shown in dashed trace. B: Illustration of the different torque profiles: black traces illustrate the step perturbations (1 Nm and 3 Nm are displayed with thin traces), ramp-down perturbations in red and ramp-up perturbations in blue. C: Schematic representation of the effect of a ramp-down perturbation profile on the state estimation: the overestimation (gray region) result from the difference between the expected perturbation profile (step-function, dashed) and the actual perturbation (solid). The ramp-down profile was designed to produce an overestimation as illustrated in Figure 1 C. The opposite reasoning applies to ramp up-perturbation that produces an underestimation of the present state of the joint.

**Figure 4:**
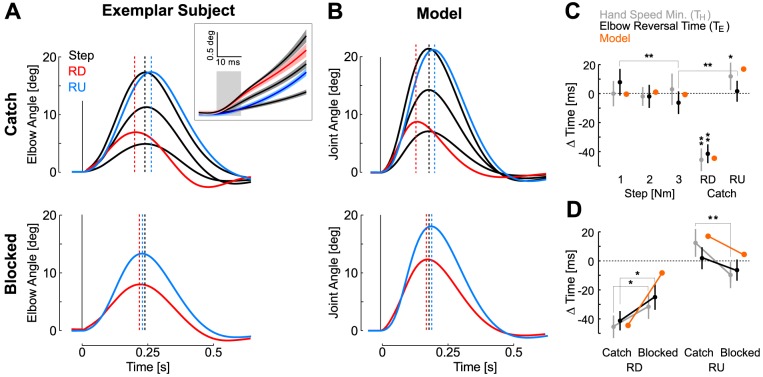
Experiment 1: Behaviour. A: Average elbow motion from one exemplar subject when step perturbations were expected (top) and when ramp-profiles were presented in blocked manner (bottom). Displays use the same color code as in Figure 3 B. The three black traces correspond to 1 Nm, 2 Nm and 3 Nm step-torques. The vertical black dashed line is the reversal time averaged across responses to all step-torque perturbations. Blue and red dashed lines represent the reversal times of ramp-up and ramp-down perturbations. The inset shows the initial elbow displacement for the different perturbation profiles and magnitude (average ± SD across subjects). The gray rectangle emphasizes that the initial joint displacement across ramp-up (down) perturbations and the 1 Nm (3 Nm) step were similar for the first ∼10 ms. B: Same as A from the model simulations. C: Timing of the corrective response measured in joint coordinates based on the elbow reversal time (T_E_, black) and in Cartesian coordinate based on the first hand speed minimum (T_H_, gray) for each perturbation profile (1, 2 and 3 Nm step torques; RD, ramp-down; RU, ramp-up; mean ± SD across subject). Orange dots represent the joint reversal time computed from model simulation. Times were measured for each subject and simulations relative to the mean across responses to the step torque perturbations. Single (double) star(s) indicate significant differences at the level *P*<0.05 (*P*<0.01). D: Effect of context on the timing of the corrective response for the ramp-down and ramp-up torque profiles with the same color code as in panel A.

We tested the hypothesis that the brain uses sensory prediction to drive the motor response by exposing participants to a large number of step torque perturbations (1 Nm, 2 Nm and 3 Nm, see Methods), of which typical evoked motion is depicted in Figure 3 A. Different directions and magnitudes were used to ensure that participants were expecting a step profile, regardless of the step amplitude. The effect of unexpected amplitude changes is thoroughly addressed below (see Experiment 4). We used ramp-up and ramp-down perturbations as catch trials in order to induce unexpected variations in the joint displacement (Figure 3 B). We reasoned that if the perturbation has a ramp-down or ramp-up profile while a step torque is expected, the prediction based on sensory feedback would lead to over- or under-estimation respectively, which should be expressed in the motor response (as illustrated in Figure 3 C for ramp-down profiles). Elbow displacements are illustrated in Figure 4 A: notable is the variation in the time of the peak elbow displacement (dashed vertical lines) following ramp profiles (ramp-up, red; ramp-down, blue) relative to those of step torque perturbations (black traces). The inset in Figure 4 A shows for all subjects that the initial joint displacement for the first ∼10 ms following ramp-down or ramp-up profiles corresponds to the 3 Nm and 1 Nm step perturbations, respectively (gray rectangle, inset). Therefore, readouts of the initial limb motion do not permit to determine whether the underlying torque is a ramp-up (ramp-down) or a step perturbation (3 Nm or 1 Nm), inducing errors in state estimation at the onset of the motor response.

We found that the reversal time was a sensitive parameter that captured the effect of the profile on the kinematics of the corrective movements as well as the modulation of the feedback responses across contexts (catch or blocked, see Methods). We measured this parameter in joint coordinates as the time of maximum elbow displacement, or in Cartesian coordinates as the time of the first hand-speed minimum. Overall, we found that there was no main effect of the step magnitude on the reversal times and hand speed minimum (one-way ANOVA across step magnitudes, *F*<0.65, *P*>0.1). A closer look revealed a significant difference between reversal times following 1 Nm and 3 Nm perturbations (paired t-test, t_(12)_ = 3.6, *P*<0.01). This trend was not observed for the timing of hand speed minimum. The effect of the profile on the reversal time was robust and independent of the coordinate system: both elbow reversal time and hand speed minimum following ramp-down occurred significantly earlier than those of step torque responses regardless of the amplitude of the step (Figure 4 C, t_(11)_>6.5, *P*<0.001). The opposite effect was observed following ramp-up profiles, with a significant increase for the time of hand-speed minimum relative to those of step-torque responses (t_(11)_>2.1, *P*<0.05), and a significant increase in elbow reversal time relative to 3 Nm step torque responses (t_(11)_ = 3.03, *P*<0.01).

Importantly, the changes in reversal times observed in Figure 4 C are not a simple consequence of physics and of the time-varying ramp profiles. Instead, these changes reflect that participants relied on a feedback control strategy that depended on the context. When participants had to counter the same ramp-up or ramp-down torques presented in a blocked manner, they altered their feedback responses and the timing of corrective movements shifted towards the values previously measured for step torque profiles (Figure 4 A, bottom and 4 D). Following a ramp-down perturbation, both elbow reversal times and times of hand-speed minimum significantly increased towards values corresponding to step torques (t_(11)_>1.88, *P*<0.05). For the ramp-up torques, the time of hand speed minimum decreased significantly (t_(11)_ = 2.95, *P*<0.01). Elbow reversal times followed the same trend (t_(11)_ = 1.54, *P* = 0.075).

**Figure 5:**
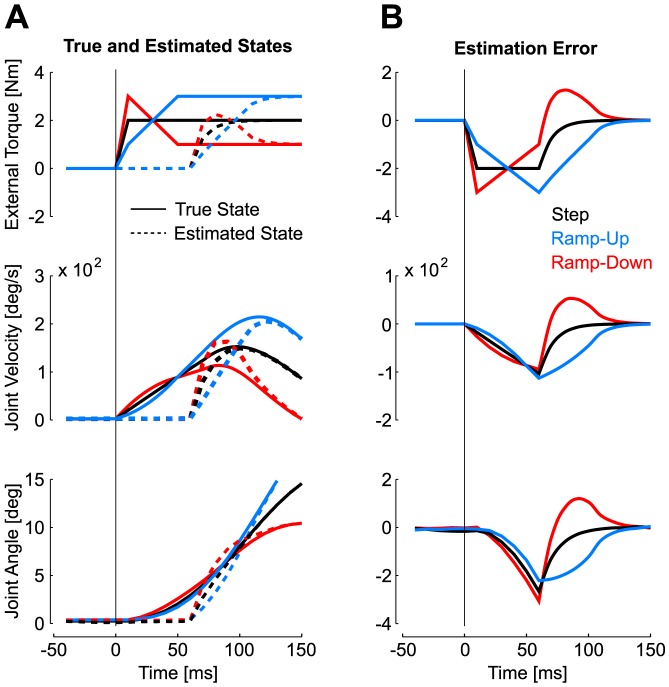
State estimation following unexpected perturbations. A: Illustration of the actual (solid) and estimated (dashed) state variables. From top to bottom, displays are the external torque (T_E_), the joint velocity and the joint displacement. 2 Nm step perturbation is represented in black, ramp-down in red and ramp-up in blue. B: Estimation error for each perturbation profile, defined as the difference between the estimated state and the true state.

The model based on Kalman filtering explains the effect of the perturbation profiles on the kinematics of the corrective movements (Figure 4 B). Prior expectations in the model were determined by the dynamics of the external torque (Methods, Eqn. 3). The time course of the actual and estimated state variables is shown in Figure 5. Under the hypothesis that the external torque is constant, the estimates of this variable can be seen as a delayed and filtered version of the actual perturbation (Figure 5 A, top). This produces an over- (under-) estimation following the ramp-down (up) perturbation as illustrated by the estimation error (Figure 5 B). These estimation errors are propagated to the other state variables, leading to an over- (under-) estimation of the actual joint velocity and displacement. These estimation errors result from the fact that the Kalman filter simultaneously corrects the present and past states under the assumption that the external torque was constant throughout the feedback delay period (δt = 60 ms). Our simulations capture three critical aspects of the data. First, the model predicts an invariant reversal time across the different values of step magnitude (Figure 4 B, C). This property is a consequence of the superposition principle of linear systems, whereby scaled amounts of perturbation-related motion result in scaling of the motor response. Our data was compatible with this prediction, except for the difference observed between reversal times following 1 Nm and 3 Nm step perturbation. This difference may reflect the limitations of the linear approximation. Second, the model also reproduces the changes in the reversal times following ramp perturbations in a way that is compatible with our experimental results (Figure 4 B, C). Third, our hypothesis of a rapid update of the state estimate accounts for the observed changes in reversal times depending on whether ramp perturbations were expected or not (catch or blocked designs, Figure 4 D): simulations were obtained by feeding the controller with exact state information after artificially delaying the response, so that reversal times following step responses were exactly matched (see Methods). The difference between reversal times of step or ramp profiles is markedly reduced when the controller can rely on perfect state estimation (Figure 4 B, bottom), and the shifts in reversal times were clearly compatible with participants' behaviour (Figure 4 D). This result is an important prediction of the model: indeed the effect of the profile on the corrective movement does not solely result from physics. Instead, they reflect the model's beliefs about the external torque and their effect on the corrective response. It is important to realize that estimation and control processes are independent in our model. Therefore, as the control policy was always the same across all simulations, we can ascribe the changes in feedback responses to the estimation algorithm.

**Figure 6:**
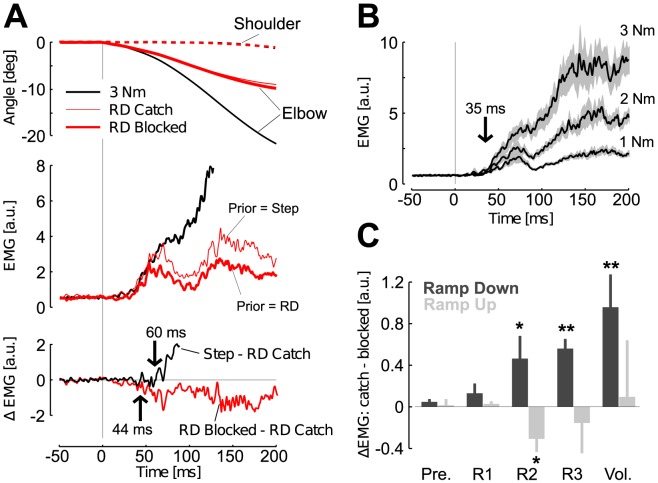
Experiment 1: Muscle responses. A: Top: Average shoulder (dashed) and elbow (solid) joint displacements following extension torques in three different cases, 3 Nm step torques (black), and ramp-down profiles in catch and blocked conditions (thin and thick red traces, respectively) averaged across subjects. Shoulder motion was identical across all conditions and the corresponding traces are superimposed. Middle: Perturbation-evoked response recorded from an elbow flexor (brachioradialis) with the same color code as in the top panel. Bottom: Difference between the 3 Nm step response and the ramp-down in the catch condition (black), and between ramp-down torques in the block and catch (red). Arrows indicate the onset of divergence from the 3 Nm evoked response. B: Response evoked by step-torque perturbations averaged across subjects. The shaded area represents the standard error. The vertical arrow depicts the latest divergence onset across all pair-wise comparisons. C: Changes in evoked response across the conditions where ramp-down (dark gray) or ramp-up (light gray) perturbations were presented in catch or blocked design. The grand average of Brachioradialis and Triceps Lateralis responses was considered for this analysis. Positive designates that the evoked activity decreased in the block condition.

We collected the activity of elbow flexors and extensor muscles in order to determine the time when prior-related components of the response influenced the feedback correction. When participants expected a ramp-down perturbation (blocked condition), the evoked response diverged from the response evoked by 3 Nm step perturbations after 44 ms for Brachioradialis (Figure 6 A, ROC Analysis) and 40 ms for Triceps Lateralis. In contrast, the same analysis revealed that in the catch condition, responses followed those evoked by 3 Nm step torques until 60 ms after perturbation onset (76 ms for Triceps Lateralis), whereas the elbow displacement was equal across catch and block conditions until >100 ms (Figure 6 A). Observe also that the shoulder did not move until >100 ms as a result of the multi-joint torque, which validates the single joint model to address the problem of state estimation following the perturbation. The onset of divergence between ramp-down responses from the 3 Nm step torque across catch and block conditions must be compared with the onset of divergence measured across the step perturbations when participants relied on adequate priors. In this case, responses diverged in less than 35 ms for Brachioradialis and Triceps Lateralis in all pair wise comparisons (Figure 6 B).

Changes in activity resulting from mistakenly tracking the corresponding step function were significant in the long-latency time window. Following ramp-down profiles, the pre-perturbation activity (−50–0 ms, see Methods), and the short latency response (R1, 20–45 ms) were not significantly different across catch and block conditions (Figure 6 C, one-tail paired t-test, t_(11)_<1.31, *P*>0.1), whereas significant context-related modulation was found in the long-latency and early voluntary epochs of time (R2, 45–75 ms: t_(11)_ = 2.04, *P* = 0.03; R3, 75–105 ms: t_(11)_ = 5.84, *P*<0.001; Vol., 120–180 ms: t_(11)_ = 2.98, *P* = 0.006). This effect means that, for similar baseline and short-latency muscle activity, the long-latency response was significantly reduced when participants were expecting a ramp-down profile. The down-regulation of the response started in the R2 time window and likely resulted from internal processing of sensory data given that the joint displacement was identical across conditions. The opposite tendency was observed following ramp-up perturbations: responses in the blocked condition displayed significant modulation in R2 (t_(11)_ = 2.34, *P* = 0.019), whereas the other epochs displayed statistically similar activity (t_(11)_<1.64, *P*>0.05). We performed an additional control experiment to address why the response modulation was smaller following the ramp-up perturbations and found that it was likely due to the relatively high perturbation magnitudes (3 Nm), generating very high response rate. We observed a stronger response modulation after reducing the perturbation loads (see Methods).

In all, the prior-related component influences the muscle response within about 60 ms of perturbation onset, in a way that correlated with changes in the expected relationship between the initial joint displacement and the state of the limb at the onset of the motor response.

### Experiment 2

A surprising result from Experiment 1 is that, on average, the difference between ramp-down responses across conditions persisted for a prolonged period of time (Figure 6 A). This suggests that the internal priors are quite strong, and that the sensory data does not fully overwrite it even after the time varying portion of the ramp-down perturbation. Given the strength of these priors in the corrective response, an important question is how rapidly they can be updated should a distinct perturbation profile be experienced. We designed the second experiment to test this prediction. We used a random adaptation paradigm and tested the influence of changes in perturbation profiles on the response to the next trial [20], [21]. This paradigm presents the advantage to test the effect of a change in the perturbation profile on a large number of trials, which is typically required for the analysis of EMG data.

**Figure 7:**
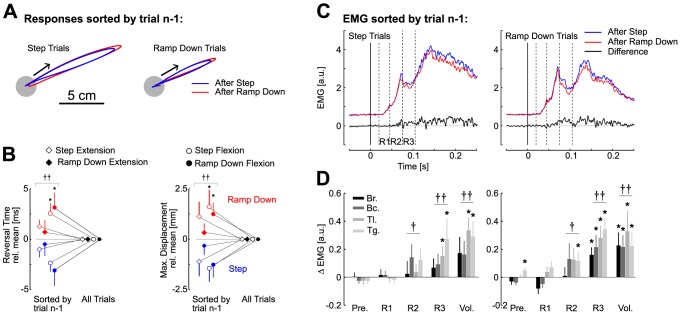
Results of experiment 2. A: Average hand path from one representative subject following step torques and ramp-down torques sorted by trial n-1. The blue traces are the average across all trials preceded by a step perturbation and the red traces are the average across all trials preceded by a ramp-down perturbation. B: Trial-by-trial modulation of the corrective response depending on the preceding trial for step (open symbols) and ramp-down (filled symbols) flexion and extension perturbations (disk and diamond, respectively). Significant differences in data from single perturbation profile (*) and from all perturbation pooled together (†) are shown at the level *P*<0.05 (one symbol) and *P*<0.01 (two symbols). C: Grand average across the four muscles of interest and across subjects of trials sorted by trial n-1 with identical color code as in panel A for step torques (left) and ramp-down (right) torque profiles. The black traces are the differential signals between blue and red responses. The dashed vertical lines represent the different epochs of rapid motor responses (see Methods). D: Binned analysis of the difference in activation across responses preceded by step torques and responses preceded by ramp-down torques for the four muscles of interests following step perturbations (left) and ramp-down perturbations (right). As in panel C, positive designates an increased activity when the trial is preceded by a step perturbation. Bars represent one standard error across the 12 subjects. From dark gray to light gray, displays are Brachioradialis (Br.), Biceps (Bc.), Triceps Lateralis (Tl.), and Triceps Long (Tg.). Stars indicate significant modulation of the corresponding muscle at the level *P*<0.05 and the dagger (†) indicates significant modulation from all muscle samples pooled together (one symbol, *P*<0.05; two symbols, *P*<0.01).

The 2 Nm step and ramp-down were chosen based on the results of the first experiment. After the habituation blocks (see Methods), the two perturbations profiles were randomly interleaved and equally likely. We sorted responses to each torque profile (step or ramp) by the preceding trial and found that the responses following a step perturbation displayed more vigorous corrections for either perturbation type (quicker reversal times and smaller total displacement) than those following a ramp-down perturbation (Figure 7 A and B). EMG responses sorted by the same criterion correlated with the trial-by-trial changes in the behavior: up- or down-regulation was observed depending on whether the preceding trial was a step or a ramp-down perturbation, respectively (Figure 7 C and D). Importantly, significant changes in muscle responses from all muscle samples pooled together were found from the onset of the R2 time window (45–75 ms, Figure 7 D), which confirms the results of Experiment 1. The difference between perturbation responses to the same profile, (step or ramp-down) depending on the previous trial was found at 66 ms, within the long-latency time window (ROC on the differential signal relative to the pre-perturbation variability). Observe that this divergence onset is found later than those measured in the first experiment because, in this case, the divergence were measured relative to the 3 Nm step responses rather than across conditions. These results emphasize that internal models of the perturbation profiles can be adjusted following the occurrence of a single unexpected perturbation profile.

### Experiment 3

**Figure 8:**
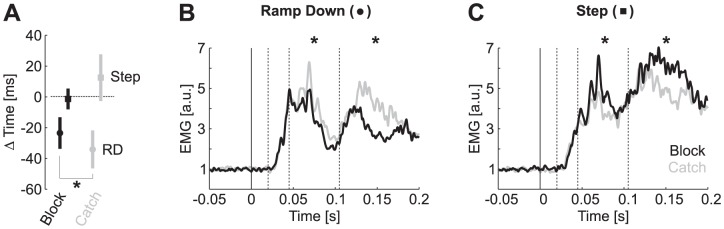
Effect of muscle pre-activation. A: Changes in reversal times relative to the average across all step responses from the block condition (similar as in Figure 4 C). Black and gray illustrate catch and block conditions while round and square items correspond to ramp-down and step responses, respectively. B: Response evoked by ramp-down perturbation when these profiles are expected (black) or presented as catch trials (gray). Data are from Brachioradialis and the trace is the average across subjects. Stars indicate that the response averaged in the corresponding time window differed significantly across catch and block conditions (*P*<0.05). C: Same as B for the responses following step perturbations (2 Nm).

We first addressed whether inverting the internal prior affected the response to the previously expected step perturbation profiles. As predicted, reversal times following step perturbations tended to be delayed when participants were expecting a ramp-down profile, although this trend was only close to significant (Figure 8 A, t_(7)_ = 1.87, *P* = 0.051). Importantly, the long-latency and early voluntary epochs displayed significant modulation across catch and blocked conditions (Figure 8 C, t_(7)_>1.9, *P*<0.05), showing that the priors used in Experiment 1 can be reversed and modulate the response to the step perturbations.

Second, this experiment was designed to investigate whether the response modulation persisted when the muscles were pre-activated. This experiment was motivated by the response differentiation found at ∼44 ms in the first experiment, which, in theory, indicates that the short-latency pathway may have contributed to the response modulation. We applied a background load on the elbow joint (−1 Nm) to evoked the same baseline activity across the two series of blocks in which ramp-down trials were presented as catch trials or in blocked fashion (Pre. across conditions, t_(7)_ = 0.4, *P* = 0.65). A short-latency response was clearly evoked by each perturbation profile (R1 versus Pre., t_(7)_>2.7, *P*<0.05), but these R1 responses were statistically similar across catch and block conditions (t_(7)_<0.4, *P*>0.25). In contrast, long-latency (45 ms–105 ms) and early voluntary responses (120 ms–180 ms) exhibited significant modulation across conditions (Figure 8 B and C, t_(7)_>1.9, *P*<0.05). The onset of divergence across conditions was found at 55 ms (ROC on the differential signal relative to the pre-perturbation activity). As in Experiment 1, the modulation of the muscle response correlated with the change in reversal time (Figure 8 A). Therefore, the modulation of long-latency responses could be reproduced with similar gains in the short-latency stretch response.

### Experiment 4

**Figure 9:**
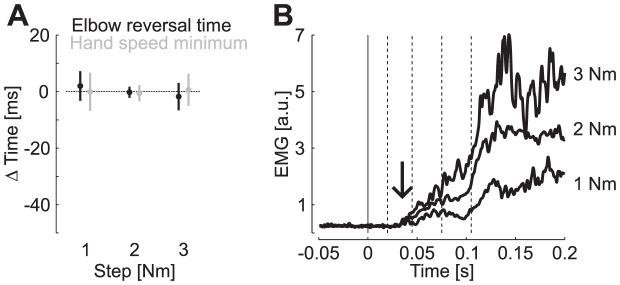
Effect of unexpected changes in perturbation magnitude. A: Reversal times and hand speed minima across the tested values of step magnitudes as presented in Figure 4 C. We used the same scale as in Figure 4 C to emphasize that unexpected changes in step magnitude could not account for the effect of ramp profiles on the times of reversal or hand speed minima. B: Perturbation evoked response from Brachioradialis averaged across subjects. The vertical arrows illustrate the latest onset of divergence across all pair wise comparisons based on ROC analysis (35 ms).

In this experiment, we verified that the effect reported above was specifically related to the perturbation profiles independent of their magnitude. In theory, the controller only needs to know the perturbation profile to correct the state estimate, independently from the perturbation magnitude. A direct prediction of the model is that participants expecting a step torque should be able to respond to any perturbation magnitude provided that it follows a step function. Alternatively, if changes in control gains are involved, we expect to see a delayed corrective movement following the unexpected 3 Nm step torques since subjects were expecting a smaller perturbation (2 Nm). Feedback responses should also overcompensate for an unexpected 1 Nm perturbation. We tested these predictions by exposing participants to a large number of step torques of 2 Nm and presented step perturbations of 1 Nm or 3 Nm as catch trials following the same distribution as in the first experiment (see Methods). We found that reversal times were essentially invariant across all step magnitudes even when the large (3 Nm) and small (1 Nm) perturbations were unexpected. Figure 9 shows the reversal times and the time of hand speed minimum. As observed in Experiment 1, the reversal times displayed little variation across the different values of the step perturbation magnitude. We used the same axis as in Figure 4 C to emphasize that unexpected changes in step magnitude cannot account for the effect of unexpected ramp-profiles on the reversal times. Indeed, the variation in reversal times evoked by ramp-down torques are of the order of −40 ms on average (Figure 4 C), which is clearly outside of the range of values reported in Figure 9 A. While the effect of ramp-up torques was overall smaller, the shift in reversal time of ∼10 ms on average (Figure 4 C) is also outside of the range reported in this experiment.

These results suggest that the variation in the kinematics of corrective movements emphasized above is specific to the shape of the perturbation. Muscle responses of an elbow flexor are shown in Figure 9 B: the scaling of the response with the magnitude of the step can be observed very early. The measured onset of divergence across all paired comparisons of response populations was found in the short-latency time window (ROC, 35 ms vertical arrow). This result shows that although changes in magnitude were unexpected, participants did not track any inadequate response strategy as observed following ramp perturbations.

## Discussion

This study shows that internal models of the perturbation loads influence long-latency responses to mechanical perturbations. Simulations based on optimal feedback control suggest that these priors reflect a rapid correction of the estimated state of the limb based on sensory prediction. In general, internal priors strongly influence decisional processes [22], [23], multi-sensory integration [24]–[27] and forward predictions [12], [16], [17], [28]. This study shows that internal priors also influence the feedback control strategies following mechanical perturbations.

Although previous studies have suggested that the brain uses sensory prediction following a perturbation [13], [14], [29], direct evidence was missing because the latter studies addressed changes in feedback responses over longer time windows (>100 ms), during which the usual forward dynamic model is engaged (Figure 2, Motor Prediction). Also, these studies did not investigate how quickly the prediction performed on sensory signals was used to guide motor responses. In order to disambiguate sensory from motor prediction mechanisms, it was necessary to manipulate the perturbation over a time window during which the motor command does not influence the motion of the limb. We addressed this concern by varying the load profiles over a time window corresponding to the shortest sensorimotor delays, as we suspected that the sophistication of long-latency responses is at least partially due to a rapid update in state estimation [30].

Our approach focuses on the rather simple case of a constant external torque, which is easy to model in the framework of linear systems. However, the limitations of linear systems are only theoretical and our data suggest that participants were able to learn more complex priors corresponding to non-linear ramp-up or ramp-down perturbations. Whether we are able to learn any perturbation profile, or equivalently any mapping between the sensed initial motion and the actual state of the limb, is an open question. Another important question is how multiple priors can be acquired. Our daily lives suggest that we can acquire motor skills in distinct tasks (such as biking and skating) without re-learning every time that we switch between tasks. A recent study in the context of force field learning has emphasized that multiple internal models can be acquired provided that the internal representation of the movements are distinct [31]. If a similar mechanism underlies internal models for sensory predictions, we expect that contextual factors play a key role for the acquisition of multiple priors associated with distinct motor tasks.

Overall, the effects of prior expectations on the muscle response as well as on the kinematics of the corrective movements were quite small. This is not surprising as perturbations were manipulated over a very short time interval (∼50 ms), and the resulting unexpected change in limb motion can only be small. A clear difficulty is that it is not possible to investigate the case where no estimation at all is engaged in the response. Instead, we had to manipulate participants' expectations to extract the evidence for a sensory predictor. Although our approach evoked small effects in terms of magnitude, the results were consistently reproduced across experiments. Importantly, we also showed with simulations that ignoring the use of sensory predictions could lead to instability that should clearly be avoided at all cost.

We also demonstrate two key properties of the sensory predictor. First, we show that the influence of a prior during mechanical perturbations occurs from ∼45 ms to ∼60 ms, at which time the motor response started to diverge towards the appropriate profile. Assuming a contribution of the transcortical feedback with sensory and motor delays of about 30 ms [32], [33], it is possible that the internal prior uses at most 15 ms of sensory information. Accumulating sensory evidence overrides this prior with a further 15 ms of information. However, we found that the responses remained biased by the expected profile well beyond this early time period, which may reflect the continued influence of the prior.

A second key property of sensory predictors is that it is modified on a trial-by-trial basis, which parallels the properties of the voluntary motor system observed in force-field learning studies [20], [34]. We randomly interleaved two response profiles and found that perturbation responses were also modified by the perturbation applied on the previous trial. This result emphasizes that similar mechanisms underlie voluntary control and rapid feedback responses to perturbations [30].

In principle, it is also possible that feedback gains were changed independently from any update in state estimation. Such changes in feedback gains may originate from internal set of the control strategy, or from changes in the peripheral motor apparatus through co-contraction and stiffness modulation [35]–[37]. While it is difficult to completely rule out such alternative interpretation, we believe that, in the present case, several features of our data argue against non-specific changes in control gain. First, we showed that applying control gains to delayed sensory feedback was likely to generate unstable oscillations. Although the control performances in such cases should be thoroughly investigated, our simulations suggest that delays on the order of tens of milliseconds cannot be ignored to produce fast and accurate feedback responses (see Figure 2).

Second, we found that the modulation of long-latency responses according to prior expectation was present even after controlling for the pre-perturbation activity and short-latency reflex (Experiment 3). This experiment was partially motivated by the divergence onset between the expected ramp-down from the 3 Nm perturbations that we found at the end of the short-latency time window (Experiment 1). However, even with similar R1 responses, it is possible that rapid sensory predictions occurred at the periphery [38], and that the sensitivity of the spindles to changes is muscle velocity and acceleration was adjusted according to participants' expectations [39]–[41]. Besides possible adjustments of the peripheral apparatus, our suggestion is that a similar sensory input is mapped into a distinct motor output as a result of a learned relationship between the initial joint displacement and the state of the limb. An important question is to determine under which circumstances motor systems rely on non-specific modulation of the short latency pathway as opposed to a novel sensorimotor mapping.

Finally, unexpected changes in the step magnitude did not generate any over nor under compensation. Responses to 1 Nm and 3 Nm step perturbations were clearly similar regardless of whether changes in perturbation magnitudes were expected or not. Therefore, changes in reversal times evoked by ramp perturbations could not be explained by a possible modulation of control gains involved in response to unexpected changes in perturbation magnitude. These results were predicted by the model: the Kalman filter can correct the present estimate of the state of the limb by combining the sensed step magnitude of each individual trial with prior assumptions about the perturbation profile. As a consequence, time-varying feedback responses result from a constant feedback control policy applied to time varying estimates of the state of the limb, which does not require any prior knowledge about the perturbation magnitude. The controller only needs to know the perturbation profile.

Future studies should investigate the underlying neural pathway. The latency of the prior-related component already sets physiological constraints on the possible candidates. The cerebellum is clearly a candidate region given its known implication in prediction processes associated with descending commands [16], [42]–[44]. Our sensory-based prediction is similar in many respects; the main differences are that sensory information is used as input rather than the motor command, and the time interval over which the prediction is computed is distinct. Otherwise, these two prediction processes need the same internal model of limb dynamics. The cerebellum also responds to mechanical perturbations in the required time window [45], [46] and projects to the primary motor cortex that is known to contribute to long-latency activity [47]–[49]. In addition, cerebellar dysfunction induces oscillatory feedback responses to perturbations [50]–[52], which recalls the stability issue encountered when feedback delays were ignored (Figure 3). From this perspective, cerebellar modulation of reflex gains could be a stabilizing mechanism that anticipates what the motor system should do in the present time.

A sensory prediction is critical when abrupt perturbations induce large displacement as in the present study. However, disturbances can also be encountered at smaller scales including noise in neural circuits, and feedback responses are likely engaged at the level of small deviations corresponding to natural variability [53]. Even small deviations in the limb motion must be processed to accurately adjust the ongoing motor command. In this respect, the sensory predictor must be engaged during voluntary movements as well as following external perturbations. Motor learning and development of motor skills is also clearly contingent upon the acquisition of both sensory and motor predictive models since feed-forward and feedback processes must incorporate knowledge of the dynamical interaction with the environment [14]. Biking on a bumpy road, skating or countering wind gusts pushing one's sail are examples of tasks that we could hardly learn to stabilize without adaptive sensory prediction of the state of the body.

## Methods

### Ethics Statement

The Queen's University Research Ethics Board approved the experimental protocol and participants gave written informed consent following standard procedures.

### Apparatus

Subjects interacted with a virtual reality display showing visual targets and a right-hand aligned cursor in the horizontal plane. Participants' right arm was placed on an exoskeleton that can selectively apply torques at the shoulder and/or elbow joints (KINARM, BKIN Technologies, Kingston, ON [54], [55]). Arm motion was constrained to the horizontal plane. The target (radius 1.2 cm) was located at 45 and 90 degrees of shoulder and elbow angles for each subject (Figure 3 A). Perturbations were applied after a random delay (between 1 s and 2 s) following stabilization at the start target. In all cases, perturbations were built up in 5 ms and equal amounts of torque were applied at the shoulder and elbow joints. This procedure allows compensating for interaction torques at the elbow joint, which cancels the initial shoulder acceleration and produces pure elbow motion for ∼150 ms [56]. The hand-aligned cursor was extinguished at perturbation onset. Participants were instructed to return to the target within 800 ms of perturbation onset and stabilize for 2 s. We used different time varying perturbation profiles to produce an ambiguous relationship between the present state of the limb and the initial joint displacement sensed after the feedback time delay. The different perturbation profiles are illustrated in Figure 3 B. The step perturbations of different magnitudes followed a linear buildup of 5 ms. The ramp-down perturbation followed a linear ramp from 0 Nm to 3 Nm in 5 ms, and then from 3 Nm to 1 Nm in 50 ms. The ramp-up perturbation followed a linear build up from 0 to 1 Nm in 5 ms, followed by a second linear build up from 1 Nm to 3 Nm in 50 ms (Figure 3 B).

### Main Experiments

#### Experiment 1

This experiment tested whether feedback responses to perturbation engaged internal priors about the perturbation profile. To do so, we used step torque perturbation of varying size and direction so that participants were expecting this perturbation profile. We addressed the effect of unexpected time-varying perturbation by using ramp-up or ramp-down perturbations randomly presented as catch trials (Figure 3 B). Subjects (N = 12) completed four identical blocks separated by short pauses to avoid fatigue. Each block consisted of 60 step trials (10 flexion or extension ×1, 2 or 3 Nm), 8 ramp-up trials and 8 ramp-down trials (4× flexion or extension for each profile), summing to a total of 76 trials per block. We also investigated whether participants altered their responses to each of the ramp profiles if they were expected. After the four initial blocks, participants were exposed to two blocks of ramp-up or ramp-down perturbations presented in blocked fashion (60 trials, 30× flexion or extension for each perturbation profile) in order to test whether changes in the context modulated the feedback response to these perturbation profiles.

We also performed an additional experiment to address the influence of the load magnitude on the response profiles to the ramp-up perturbations. Indeed, the response modulation following ramp-up perturbations was weaker, which is partially due to the large perturbation loads applied (3 Nm). We used the same paradigm on 8 participants while using load magnitudes reduced by 20% in order to see whether smaller load magnitudes leave more room for the response modulation following the ramp-up perturbations. Participants countered step torques of 0.8 Nm, 1.6 Nm or 2.4 Nm while ramp-up perturbations (from 0 to 0.8 Nm in 5 ms, and 0.8 to 2.4 in 50 ms) were presented as catch trials with the same distribution as in the main experiment. Participants were also exposed to a block of ramp-up perturbations. The sequence of blocks was randomized across participants. This control experiment reproduced the results of the main experiment and amplified the response modulation across conditions (see Results). We found a stronger effect following ramp-up torques: the reversal times were significantly delayed relative to those following step perturbations (ΔTime = 11.5 ms on average, t_(7)_>2.3, *P*<0.05), and the modulation of muscle response was significant in the R2 (t_(7)_ = 2.21, *P* = 0.031) and early voluntary time windows (t_(7)_ = 2.02, *P* = 0.041).

#### Experiment 2

In this experiment, we investigated the effect of a change in the perturbation profile on the feedback response strategy of the following trial by using a random adaptation paradigm. This experiment sought to examine whether the component of the rapid feedback responses that depends on prior expectations can be quickly adjusted from trial-to-trial as observed for voluntary control [20], [34]. To do so, we chose step torques (2 Nm) and ramp-down profiles based on the results of Experiment 1 as these profiles elicited a robust behavioral effect. After performing a series of 60 of each perturbation type to familiarize subjects with the experimental setup, subjects (N = 12) were exposed to four blocks in which step or ramp-down torques were randomly interleaved and equally likely. Each block consisted of 60 trials including ramp-down and step perturbations (15× step or ramp-down×flexion or extension). The order of the initial habituation blocks was randomized across participants to avoid inducing a systematic bias towards the profile experienced in the last habituation block.

### Control Experiments

#### Experiment 3

This experiment tested two main effects. First, we used a similar paradigm as in Experiment 1 except that we had step perturbations as catch trials while participants were expecting a ramp-down perturbation. Second, this experiment was performed with a constant load applied on the elbow (−1 Nm) to in order to control for the pre-perturbation activity and short-latency reflex. In one series of blocks, participants (N = 8) were instructed to counter the perturbations including step torques of ±1 Nm, ±2 Nm and ±3 Nm. Perturbations were randomly interleaved and added to the constant background load. Ramp-down perturbations were presented as catch trials following the same distribution as in Experiment 1 (16 ramp-down perturbations for 60 step perturbations). In another series of blocks, ramp-down perturbations were blocked and 2 Nm step-torques were presented as catch trials. The sequence of each series of blocks was varied across subjects to eliminate possible order effects.

#### Experiment 4

We finally examined the effect changes in control gains evoked by an unexpected step magnitude. We needed to verify that unexpected changes in the step magnitude did not produce variation in movement kinematics that could account for the effect emphasized in the first experiment. Participants (N = 8) had to counter step torques of ±2 Nm presented in blocks of 48 trials (24× flexion or extension). Step perturbation of ±3 Nm and ±1 Nm were presented as catch trials (4×3 Nm or 1 Nm×flexion or extension per block), summing to a total of 64 trials per block. Each subject performed three blocks.

### Data Collection and Analysis

Shoulder and elbow motion were collected at 1 kHz and digitally filtered at 50 Hz (4^th^ order dual-pass Butterworth filter). We considered both the kinematics of elbow motion as well as hand paths in Cartesian coordinates to validate the use of the single joint model presented below. Muscle activity was collected by means of surface electrodes attached on the muscle belly after light abrasion of the skin with alcohol (DE-2.1, Delsys, Boston, MA). We concentrated on the mono-articular elbow muscles for Experiment 1, 3 and 4 (Brachioradialis, Br.; Triceps Lateralis, Tl.), and on the mono- and bi-articular elbow muscles for Experiment 2 (Biceps, Bc; and Triceps Long, Tg., in addition to Br. and Tl.). The raw EMG signal was amplified (gain = 10^4^), digitally band-pass filtered (10–400 Hz), rectified, and averaged across trials. EMG signals were normalized to the average activity measured against a 2 Nm background load for all muscle samples (except in Experiment 3 where we used the activity evoked by the 1 Nm background load), while participants maintained postural control in the initial joint configuration (elbow = 90 deg and shoulder = 45 deg). The binned analysis of muscles activity was based on average EMG across the different epochs following classical definitions (Pre., −50 to 0 ms, R1, 20 to 45 ms; R2, 45 to 75 ms; R3, 75 to 105 ms and early voluntary from 120 to 180 ms [57]). Statistical comparisons of kinematics or integrated EMG were based on one-tailed paired t-tests across the different conditions. We used Receiver Operating Characteristics (ROC) to determine the onset of divergence between time series of EMG signals [58].

### Model

The importance of the model is to provide a rationale for the experimental design as well as predictions about the effect of the perturbation profile on the kinematics of the corrective movement. The hypothesis that the brain uses a process similar to a Kalman filter was found to be a very powerful approach to characterize the online combination of internal priors with multisensory information [25], [26], [59]. We used this model in the context of optimal control to emphasize the consequences of feedback delays within a framework that is compatible with current approaches in sensorimotor control.

We considered the angular motion of a rigid body as a model of the elbow joint. The choice of a single joint model was compatible with the perturbation-related motion immediately after the perturbation onset. Indeed, because we applied similar amounts of torque at the shoulder and elbow, the initial shoulder acceleration is zero as a result of the initial joint configuration and dynamics. Our data confirmed this property as the shoulder did not move until >100 ms following the perturbation. Therefore, the problem of state estimation following the perturbation reduces to the estimation of the elbow joint displacement in agreement with the single joint model. In addition, more complex models (e.g. nonlinear models including inter-segmental dynamics) are not necessary because the single-joint model captures the problem caused by feedback delays. Thus, we kept the model as simple as possible.

The differential equation of the joint motion was coupled with a first order, low-pass model of muscle dynamics linking the control variable to the muscular torque. The net torque was the sum of a viscous torque proportional to the angular velocity, a controlled torque (*T_C_*) and an external torque (*T_E_*). The different parameters (inertia, viscosity and time constants) were estimated from physiological models [60], [61]. The controlled torque was a first order, low-pass response to the control variable (*u*) with time constant τ = 60 ms. The inertia (*I* = 0.065 Kg m^2^) was estimated from the robot structure and average anthropometric data. The viscous constant was set to *G* = 0.05 N/s. The angular motion of the joint is described by the following system of differential equations (*θ* is the joint angle and the dot represents time derivative):
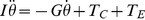
(1)

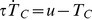
(2)

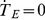
(3)


This system was transformed into a discrete time control system by using classical Euler integration with 10 ms time step in order to take noise disturbances into account. Feedback delays were set to 60 ms. This value of feedback delay is compatible with the long-latency transmission delays, and also takes into account the fact that the controller, unlike EMG, can change the control value instantaneously. We therefore added on time step to the usual ∼50 ms considered for long latency delays in order to generate more realistic simulations. The state vector is composed of the joint angle, the joint velocity, the torques and the target location (noted θ*) at each time step:
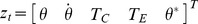
(4)The dependency of the state variables on time was omitted for clarity. In order to take feedback delays into account, the state vector must be augmented to include the previous time steps until the first time step observable by the controller. We define the augmented state as follows:
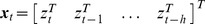
(5)where *h* = 6 represents the feedback delay expressed in number of sample times (60 ms). After reduction to the non-delayed case by system augmentation (Equation 5), the discrete dynamics and feedback can be written as:
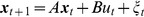
(6)

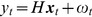
(7)The matrices *A* and *B* are determined by the system dynamics and augmentation (Eqns. 1–3), and *H* expresses that only the most delayed time-step of the augmented state vector is observable by the controller (*O_n_* and *I_n_* are zeros and identity matrices of appropriate dimension):
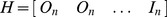
(8)We considered additive Gaussian noise (*ξ_t_* and *ω_t_*) affecting the control and feedback signals to ensure that the state estimation was independent from the control mechanism [62]. However, all simulated results were similar in the presence of signal-dependent noise. The motor noise (*ξ_t_*) only affected the control signal (Equation 2) while the feedback noise (*ω_t_*) affected all entries of the observed state vector (Equation 7).

For this class of system, the Kalman filter gives an unbiased estimate of the state vector (Equation 5) that minimizes the estimation variance [63]. The state estimation is performed in two steps. We used 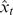 to designate the estimated state at time step *t* following standard notations. First, a prior estimate is computed based on the motor commands and internal models of the systems dynamics (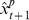). This prior estimate was also corrupted by additive Gaussian noise (*ζ_t_*):
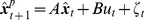
(9)Then, the prior estimate is corrected by the difference between expected and actual sensory feedback, weighted by the Kalman gain:
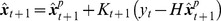
(10)The rapid update of state estimation results from the definition of the augmented state. Indeed, the second term in Equation 10 corrects the prior estimate (Equation 9), which itself contains the past state vectors (Equation 5). Hence, the Kalman filter simultaneously corrects the sequence of joint angle, joint velocities and torques over the time interval corresponding to the feedback delay. Because a constant external torque is assumed (Equation 3), the controller treated changes in the external torque as step function. Hence, the sensory prediction results from the estimation of the augmented state vector (Equation 5), under the hypothesis that the external torque was constant. The consequences of assuming an external torque on the state estimation following the perturbation is illustrated in Figure 5.

The task of the controller was to stabilize the joint at a given angle against the external torque and noise disturbances. The cost-function that penalized deviation from the prescribed joint angle was:
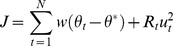
(11)
*N* is the time horizon expressed in number of time steps; *w* and *R_t_*, *t<N*, are constant scaling parameters and *R_N_* = 0. This cost-function simply penalizes deviation from θ* at minimum motor cost. For this class of control problems, the optimal control sequence is a linear function of the state estimate that can be written as follows:
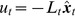
(12)All noise parameters were Gaussian with zero mean and variance equal to 10^−6^. This small value of noise variance is due to the fact that random disturbances are generated at each time step, and the variance should therefore scale according to the magnitude of the time step. When the process is simulated, we obtained a standard deviation of the joint angle of ∼0.1 deg over a 100 ms time window, which is compatible with the natural variability of unperturbed postural control [64]. The cost parameters were adjusted to match the perturbation related motion across simulations and data. Changing these parameters, as well as the delay in the feedback loop, had qualitatively no impact on the simulation results. The full control algorithm consisted in applying optimal feedback gains to estimates of the system state obtained from adaptive Kalman filter (Eqn. 12). The derivation of optimal feedback gains and Kalman gains followed procedures fully described earlier [62], [64], [65].

### Simulations

The simulations of reaching movements presented in Figure 2 were obtained by letting the system free to move for 600 ms (*w* = 0 in Eqn. 11), and then penalizing deviations from the prescribed joint angle (10, 15 or 20 deg) for 400 ms. Regarding simulations of postural control with perturbations, we used a time horizon that was sufficiently large so that the feedback gains (*L_t_* in Eqn. 12) were constant, approximating a steady-state postural control task. The different perturbation profiles were reproduced by changing the value of the external torque numerically (*T_E_*). We tested whether the forward update in state estimation could be ignored by implementing a Kalman filter with the following feedback signal instead of Equation 7:
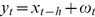
(13)where *x_t_* was defined in Equation 4. In fact, ignoring the system augmentation violates the assumption that the Kalman filter uses the conditional distribution of the feedback signal given the present state [63], and the control design is therefore prone to instability as a consequence of time delays in the feedback loop.

Finally, the blocked condition for the ramp-up/down perturbation profiles was simulated based on the assumption that the ideal control performance would be achieved if the controller could rely on perfect state information. To approximate this, we artificially set the control signal to 0 for a time interval corresponding to the feedback delay following the perturbation, and then applied the feedback gains to the true state of the system. In this case, the perfect state information corresponds to an estimation error that is zero, and the performance of the resulting control process corresponds to the best-case scenario. The artificial delaying of the response was used to generate a realistic displacement of the joint following the perturbation. We verified that the reversal times following step perturbations were identical with artificially delaying of the response, allowing us to compare changes in reversal times following ramp-perturbations. We should emphasize that the simulations based on perfect state information indicate what the system should do in the ideal case, without dealing explicitly with more complex priors. A theoretical limitation is that such complex profiles are difficult to reproduce within the framework of linear systems without additional dimensions and parameters. We performed additional simulations in which the external torque follows linear profiles (by setting the derivative of T_E_ to a non-zero value), and found the same results as with perfect state information. We decided to concentrate on the simulations with veridical state information because it provided the same prediction with fewer assumptions.

In general, the variability in the reversal times from the simulations was lower than variability observed experimentally. The confidence interval was further reduced by considering the average reversal times across 50 simulation runs. In order to emphasize that effect of the estimation algorithm on corrective movements, we did not attempt to reproduce the experimental variability and chose to concentrate on the average reversal times across simulations (Figure 4).

A shortcoming of our approach is that we change the value of the external torque (*T_E_*) during the simulations, while the feedback gains and Kalman gains depend on the initial condition (and uncentered covariance matrices) for which *T_E_* was set to 0. However, this procedure has no impact on the simulation results because we only used additive noise, making the process variability independent from the values of the state variables. In the presence of signal dependent noise, small changes in control gains and Kalman gains were observed following changes in the external torque value because higher motor commands induced more variable control signals. However, this small reduction in gains did not impact the simulation results presented above.
